# Does additional extracorporeal shock wave therapy improve the effect of isolated percutaneous radiofrequency coblation in patients with insertional Achilles tendinopathy? Study protocol for a randomized controlled clinical trial

**DOI:** 10.1186/s13063-022-06847-z

**Published:** 2022-11-07

**Authors:** Yu-Jie Song, Wen-Kai Xuan, Ying-Hui Hua

**Affiliations:** Department of Sports Medicine, Huashan Hospital, Fudan University, No.12 Urumqi Middle Rd., Shanghai, 200040 China

**Keywords:** Achilles tendinopathy, Percutaneous radiofrequency coblation, Extracorporeal shockwave therapy, Randomized controlled trial, Protocol

## Abstract

**Background:**

No conclusive evidence recommends a prior treatment for insertional Achilles tendinopathy (IAT). It is theorized that both percutaneous radiofrequency coblation and extracorporeal shockwave therapy (ESWT) relieve pain within the insertion. However, no clinical evidence shows that either treatment promotes the regeneration of the tendon or if the combination of these 2 interventions offers better function and less pain than one therapy.

**Methods:**

The study is a randomized, controlled trial with patients allocated in a 1:1 ratio to one of two parallel groups. Patients with insertional Achilles tendinopathy who are not satisfied with the effect of conservative treatment will be screened. A minimum of 38 patients will be enrolled after deciding to participate in the trial on an informed basis. Then the intervention group and the control group perform radial ESWT and sham-ESWT respectively at 6 months after percutaneous radiofrequency coblation. The primary outcome will be the Victorian Institute of Sports Assessment Achilles (VISA-A) Score. Secondary outcome measures will be Foot and Ankle Outcome Score (FAOS) scale, visual analog scale (VAS), Tegner Score, and MRI ultra-short echo time (UTE) T2* value. The assessments will occur in 6 months, 1 year, and 2 years, post-operatively. The differences between the 2 groups will be conducted as intention-to-treat basis.

**Discussion:**

We aim to investigate if radiofrequency coblation associated with ESWT can provide more encouraging imaging findings as well as functional and clinical outcomes regarding the treatment of the IAT comparing to the single radiofrequency coblation treatment.

**Trial registration:**

ChiCTR1800017898; pre-results. Registered on 20 August 2018.

**Supplementary Information:**

The online version contains supplementary material available at 10.1186/s13063-022-06847-z.

## Background

Achilles tendinopathy is a common disorder in sports and daily life. This injury is frequently encountered in middle- and long-distance runners with up to 36% incidence rate [1–3] and may cause up to 5% of professional athletes to a premature career end [4, 5]. Achilles tendinopathy is considered as a multifactorial condition whose exact etiology remains to be determined and can be divided into insertional and non-insertional tendinopathy according to its anatomical location. Insertional Achilles tendinopathy (IAT) is responsible for roughly 20–24% of all Achilles disorders [6]. Patients with IAT complain about compromised function and gradual onset of pain at the calcaneal attachment of the Achilles tendon and could be aggravated by standing, walking, and jogging.

The initial treatment is conservative management, such as eccentric exercises [7, 8], anti-inflammatory drugs (NSAIDs) [9], extracorporeal shockwave therapy [10–12], and platelet-rich plasma (PRP) therapy [13, 14]. If nonoperative treatment fails, surgery might be taken into consideration. Most surgical procedures involve debridement and decompression of Achilles tendon insertion and augmentation in case of excessive tendon loss [15]. To date, there is no gold standard treatment for IAT. Scientific evidence shows variable and inconsistent effects among different treatments [16, 17], meaning to provide a most effective management with high-quality and reliable evidence is urgently needed.

It is reported that 25 to 50% of patients with chronic tendinopathy may undergo surgery [18–20]. Percutaneous radiofrequency coblation as a minimally invasive treatment has been used in the management of lateral epicondylitis [21, 22], rotator cuff tendinopathy [23], and foot and ankle tendinosis [24]. It would reduce surgical time and facilitate an earlier return to activity. The procedure has been shown to induce degeneration of sensory nerve fibers and improves vascularity [25, 26], which, in its turn, relieves the pain and regenerates the tendon tissue, respectively. Extracorporeal shockwave therapy which is widely used in Achilles tendinopathy also shares a similar mechanism theoretically [25]. However, no clinical evidence shows those two treatments promote the regeneration and quality of the tendon.

Grosse et al. [27], Gardin et al. [28], and Juras et al. [29] reported that there was statistical significance in UTE-T2* value between magnetic resonance imaging (MRI) images of patients with IAT and normal population, which indicates that MRI UTE-T2* can be applied in clinical quantitative assessment to monitor the morphological information of the tendon and produce reliable results. In addition, the correlation between MRI UTE-T2* and clinical score can be determined.

Our aim is to evaluate the effectiveness of percutaneous radiofrequency coblation associated with an ESWT protocol and compare it to isolated radiofrequency coblation, via VISA-A score [30] primarily and MRI UTE-T2* secondarily. It is hypothesized that adjunctive ESWT will promote tendon regeneration, mitigate pain, and improve function as compared to a controlled group.

## Methods/design

The protocol will be reported in accordance with the guidelines and checklists for Standard Protocol Items: Recommendations for Interventional Trials (SPIRIT).

### Aim, design, and setting

We aim to investigate if radiofrequency coblation associated with ESWT can provide more encouraging outcomes regarding the treatment of the IAT with trustworthy evidence.

**Fig. 1:**
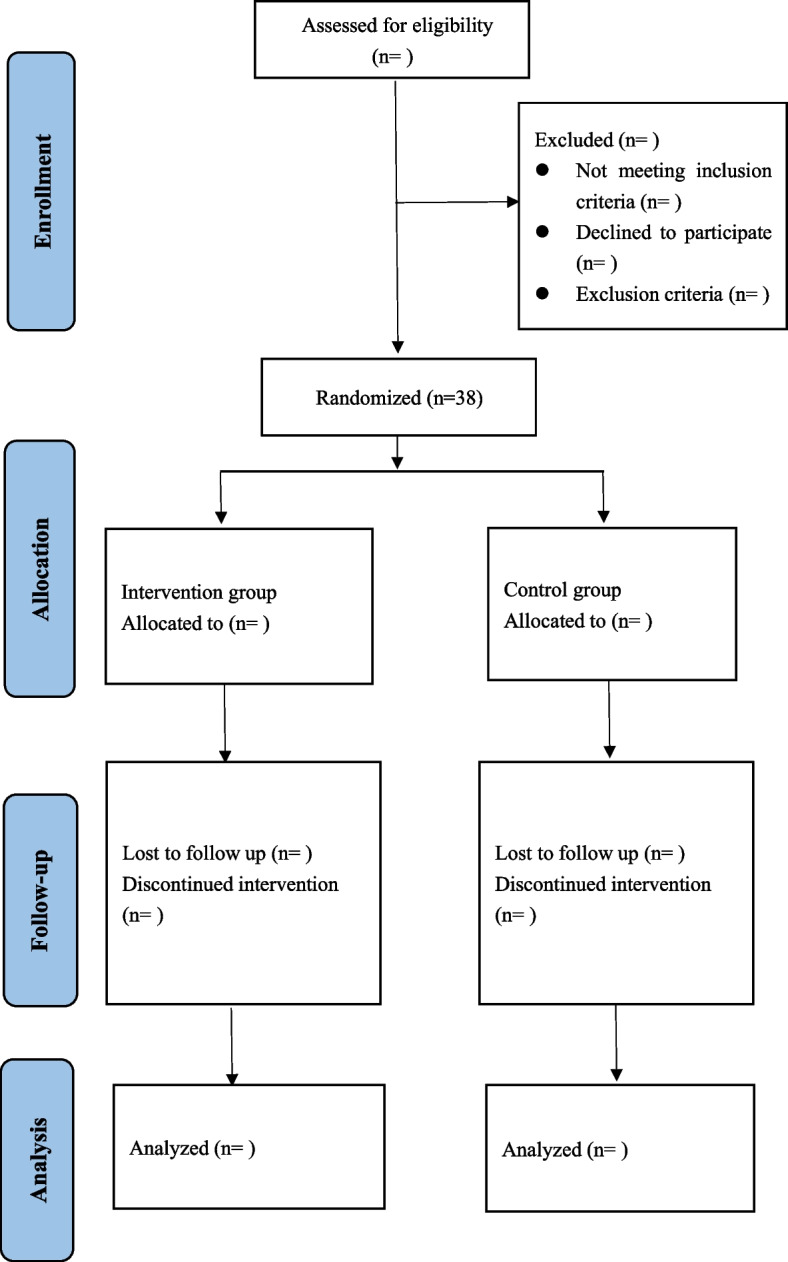
Participant flow diagram

**Fig. 2:**
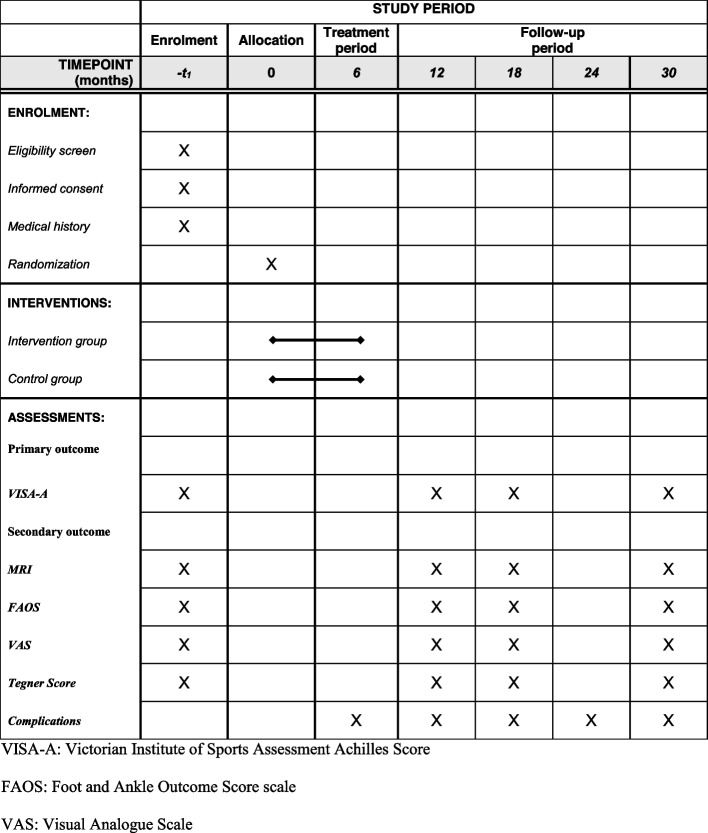
Standard Protocol Items: Recommendations for Interventional Trials (SPIRIT) Figure

This study is a randomized, double-blinded, parallel-group clinical trial (Fig. 1). The 1:1 allocation to either receive radial ESWT or sham-ESWT, after surgery is conducted using computer-generated random allocation sequences and sealed envelopes. The patients of the placebo group will also experience ESWT’s procedure, while the apparatus’ therapeutic head will be placed a support, which will block the propagation of shock waves without changing the equipment’s appearance, noise, and using process. Furthermore, the patients will feel the vibration of the machine, but the insertional region won’t be directly stimulated. The physiotherapists will not take any part in the posttreatment assessment of the patients. The outcome data will be collected by research fellows who are blinded to the treatment procedures to reduce the risk of assessment bias. The SPIRIT Figure shows in detail the schedule of enrollment and assessments (Fig. 2).

### Characteristics of participants

Patients referred to the Department of Sports Medicine, Huashan Hospital, Fudan University with insertional Achilles tendinopathy who had no symptom relief after 3 to 6 months of conservative therapy [31] other than ESWT are assessed for eligibility. The diagnosis is based on a medical history and clinical examination including persistent pain at the calcaneus tendon insertion region, and ultrasound or MRI results confirm it.

### Inclusion criteria

The inclusion criteria are as follows:

1. Medical history, clinical examination, and ultrasound or MRI results conform to the diagnosis of IAT.
2. Age 18–65 years, both genders.
3. The patient has decided to accept percutaneous radiofrequency coblation and radial ESWT.
4. The patient must be expected to be able to attend post-operation examinations.
5. The patient must be able to give informed consent.

### Exclusion criteria

The exclusion criteria are as follows:

1. Previous surgery on the affected limb.
2. The patient would not be able to strictly follow trial procedures or complete questionnaires.
3. Non-insertional or mixed tendinopathy (insertional and non-insertional).
4. Pregnancy or intention to be pregnant.
5. Currently undergoing follow-up and treatment in a psychiatry department or by other mental health professionals.

### Withdrawals

Participants may decline to continue from the trial at any time without prejudice and still encouraged to keep in touch and welcomed to inquiry and ask for necessary help.

Other reasons for discontinuation include:

1. The patient develops Achilles tendon rupture.
2. The patient develops complex or increasing regional pain.
3. Other unexpected and unpredictable circumstances when the research team judges that the risk of continuing the protocol treatment is greater than the benefit.

### Sample size

The primary outcome of this study is the VISA-A score. McCormack J et al. [32] have identified the minimum clinically important difference (MCID) of 6.5 points for the VISA-A. The study of Wei M et al. [33] indicates an expected standard deviation (SD) of 6.9 points. We have referred to the sample size calculation method of Kvalvaag E et al. [34]. To detect a difference in results between the radical ESWT and the sham-ESWT participants at a level of 5% significance and the most likely scenario for 80% power, 15 patients per group will be needed. Assuming a margin of 20% loss of primary outcome data, we propose to recruit a minimum of 38 patients in total.

### Procedures

A patient is considered to be included in the protocol if he/she meets all of the inclusion criteria and none of the exclusion criteria. Then the patient will be verbally informed of the study and given the opportunity to decide whether he/she wants to participate in the trial on an informed basis without any pressure. Then a written informed consent will be obtained from each participant. Once included, the VISA-A Score, MRI UTE T2* value, FAOS Score, VAS Score, and Tenger Score will be assessed. Within 1 week after being enrolled, the percutaneous radiofrequency coblation will be performed by an experienced surgeon. After the surgery, each patient will randomly take an unmarked, opaque, sealed envelope containing a piece of paper with a unique number from 1 to 38. Then the numbers will be divided into two sets via computing software (https://www.randomizer.org/). All patients respectively entered the intervention group and the placebo group according to their own numbers. Patients of the intervention group will receive ESWT at 6 months post-operatively. At the same time, the patients of the placebo group will also experience sham ESWT’s procedure. Either the radial ESWT or sham ESWT will be administered 4 times at weekly intervals. In all, the treatment protocols for the two groups are similar except for the intervention: radial ESWT. All the outcome measurements will be assessed again at 6 months, 1 year, and 2 years after the surgical procedure.

### Percutaneous radiofrequency coblation

The symptomatic region will be identified and marked before the anesthesia. Then, the patient would accept a general anesthetic and be placed in a prone position. A grid with a 5-mm interval will be mapped out over the marked site [35]. A 1.5-mm Kirschner wire will be used to puncture the skin and make eyelet-sized holes. The radiofrequency probe (Topaz, ArthroCare, Sunnyvale, California) will be inserted and subsequently activated for 1 second with minimum power, hole by hole. If there exists another concomitant injury such as Haglund deformity, it will be recorded and addressed by endoscopic surgery at the same time. After completion of the procedure, a standard sterile dressing would be applied to minimize tension across the wounds.

### Rehabilitation

Crutch will be recommended during the first 4 weeks after surgery. No immobilization is needed. In addition, all the patients will be encouraged to begin range of motion and isometric strength exercises immediately after surgery, and eccentric training will begin 6 weeks after surgery [36]. At the 4-week follow-up, the patients will be advised to wear regular shoe gear and continue the exercises. Patients shall be able to walk normally without any assistive device since the seventh week.

### Extracorporeal shockwave therapy

The patient will be lying on the stretcher in the prone position with barefoot, and the feet will be positioned towards the shock wave apparatus. The symptomatic area will be marked, then ultrasound gel will be applied on this region which the radial shock waves with a Dolorclast equipment (EMS Electro Medical Systems, Nyon, Switzerland), the intensity being 2000 pulses, 6–8 Hz of frequency, and 1.5–2.5 Bar of pressure per application. Compared with those of radial ESWT group, patients of the sham ESWT group will go through the same process with the same equipment, in which the apparatus’ therapeutic head will be installed a support. The support will block the propagation of shock waves without changing the equipment’s appearance, noise, and using process. Furthermore, the patients will feel the vibration of the machine, but the insertional region won’t be directly stimulated. Either the radial ESWT or sham ESWT will be administered 4 times at weekly intervals.

### MRI

Patients will be positioned feet first-supine and both feet are placed simultaneously in the dorsal part of the head coil then will be examined in a 3.0-T horizontal magnet (Discovery MR750, GE Medical System, Milwaukee, WI). For morphological evaluation of the Achilles tendon, three-dimensional fat-saturated proton-density-weighted turbo-spin echo (PDTSE) will be used. Other parameters were set as follows: repetition time (TR)=202.1 ms, field of view (FOV)=140 mm× 140 mm, slice thickness (ST) = 2 mm. Four echo time images (TE=0.032, 7.5, 20.5, and 28 ms) will be obtained. T2* value of Achilles tendon insertion region is calculated by fitting the acquired signal at different echo time to a single exponential decay model.

### Primary outcome

#### Victorian Institute of Sports Assessment Achilles (VISA-A) score

The VISA-A score is a validated questionnaire for Achilles tendinopathy and contains 8 questions assessing physical activity and pain (0, no activity/maximum pain; 100, maximum activity/no pain) [34]. And it is adapted to the local language. The Chinese version has been used in clinical trials [37, 38], proving its validity (Additional file 1).

### Secondary outcome

1. MRI: UTE-T2* value
  The MRI UTE-T2* has been reported to show significant differences between Achilles tendinopathy and normal tendon [27–29]. Thus, the morphological information of the tendon after treatment could be monitored.
2. Foot and Ankle Outcome Score (FAOS) scale
3. Visual analog scale (VAS)
4. Tegner Score
5. Complications

### Data collection

All relevant data will be collected by research fellows who are blinded to the treatment procedures and recorded in specially designed case report files. The patients will be identified by an assigned number. At the completion of the study, all identifiable data will be destroyed. The patients are informed, both verbally and in writing, that data are stored and analyzed in a computer, that the patient’s anonymity is preserved, and that the data protection legislation is adhered to.

### Adverse event management

Achilles tendon rupture, skin infections, nerve damage, and restricted ankle motion are presumably complications of both the percutaneous radiofrequency coblation and the ESWT treatment, therefore the number of complications will be registered and will be treated accordingly. Furthermore, an adverse event is defined as any unintended, unfavorable finding, symptom, or disease that occurs, whether it is considered to be related to the study or not. Adverse events will be recorded. Serious adverse events (SAEs) is defined as an event or reaction which will cause death, life-threatening situations, hospitalization or prolongation of existing hospitalization, or permanent or severe disability. SAEs will be reported to the ethics committee within 24 h.

### End of trial

The end of the trial will be defined as the collection of 2 years of outcome data from the last participant.

### Statistical analysis

All continuous data from primary and secondary outcome measurements will be summarized by descriptive statistics with the number of observations (*n*), mean, SD, minimum, median and maximum. Uncontinuous data such as co-morbidities concomitant injuries will be summarized by descriptive statistics with the number of observations, calculating the incidence separately and analyzing whether it is associated with prognosis. The primary point for analyses of efficacy will be 6 months after surgery, then 1 year and 2 years. A statistical software program (SPSS for Windows, version 23.0; SPSS Inc, Chicago, IL) will be used for statistical analysis in this study. Group comparison will be statistically analyzed by either Student’s *t* test or Mann-Whitney *U* test, depending on whether the data is normally distributed. The significance level will be set at a *p* value <0.05.

### Patient and public involvement

Neither patients nor the public was involved in the design of the study, including conceiving the research questions and deciding the outcomes. However, all participants will be asked whether they would like to receive a copy of the trial results at the time of consent. Once completed the trial, a copy of the personal results will be emailed to participants who said yes. Also, the burden of the intervention will be assessed by participants themselves using a questionnaire [39, 40].

## Discussion

Nonoperative management is the first line of treatment for IAT, though the most effective standard procedure is controversially debated [41]. However, conservative therapy is a time-consuming procedure, and approximately a quarter to half of all patients do not respond to it [20, 42]. Thus, as a minimally invasive operation, percutaneous radiofrequency coblation has been brought into attention. This technique allows patients to hasten their resolution of pain and shorten their return to activity [26, 43] though there is insufficient evidence to prove its clinical efficacy of Achilles tendinopathy, especially when the BMI of the patient is relatively high [33]. On the other hand, isolated ESWT has been reported as an alternative option for IAT by extensive studies [15, 44]. Rompe et al. [45] have showed ESWT is more beneficial in combination with eccentric exercises in non-insertional tendinopathy. Then we intend to seek for a combination method for insertional tendinopathy as well. This study protocol describes the randomized controlled trial directly comparing the effectiveness of percutaneous radiofrequency coblation associated with ESWT and isolated percutaneous radiofrequency coblation.

To our best knowledge, most studies used isolated VISA-A score as the primary outcome measurement for the prognosis of Achilles tendinopathy [46, 47] because it is the validated assessment for this quite frequent disorder. However, at the same time, there are few radiographic descriptions of the Achilles tendon. Therefore, we introduce MRI UTE-T2* as our secondary outcome measurement to monitor the status of Achilles tendon and to see the correlations with clinical scores besides the comparison of the effectiveness on function and pain between 2 interventions, which could be our secondary aim.

In conclusion, this randomized, double-blinded, parallel-group clinical trial aims to investigate if radiofrequency coblation associated with ESWT can provide more encouraging imaging findings and functional and clinical outcomes regarding the treatment of the IAT compared to the single radiofrequency coblation treatment.

## Trial status

Protocol Amendment Number: 02. Issue Date: 1 Aug 2020. The randomized trial is currently enrolling participants. Recruitment began in October 2018 and the approximate date when recruitment will be completed in October 2021. The end of the trial will be defined as the collection of 2 years of outcome data from the last participant.

## Supplementary Information

**Additional file 1:** VISA-A Chinese version.

## Data Availability

Data sharing is not applicable to this article as no datasets were generated or analyzed during the current study.

## Abbreviations

IAT: Insertional Achilles tendinopathy
ESWT: Extracorporeal shockwave therapy
VISA-A: Victorian Institute of Sports Assessment Achilles
FAOS: Foot and Ankle Outcome Score
VAS: Visual analog scale
UTE: Ultra-short echo time
PRP: Platelet-rich plasma
MRI: Magnetic resonance imaging
MCID: Minimum clinically important difference
SD: Standard deviation
TR: Repetition time
FOV: Field of view
ST: Slice thickness
SAEs: Serious adverse events
