# Supplementary material for: Does additional extracorporeal shock wave therapy improve the effect of isolated percutaneous radiofrequency coblation in patients with insertional Achilles tendinopathy? Study protocol for a randomized controlled clinical trial
Source: Trials. 2022 Nov 7;23:925. doi: 10.1186/s13063-022-06847-z (PMC9641927; doi:10.1186/s13063-022-06847-z)
Supplement: Supplementary file 1 — Additional file 1: VISA-A Chinese version. [file 13063_2022_6847_MOESM1_ESM.docx]

附表：VISA-A维多利亚学院运动功能-跟腱评分(100分制)

1.您早起后跟腱区域僵硬持续的时间？

|  |  |  |  |  |  |  |  |  |  |  |
| --- | --- | --- | --- | --- | --- | --- | --- | --- | --- | --- |

100分钟 0分钟

0 1 2 3 4 5 6 7 8 9 10

2. 当您处于热身后的状态，在台阶上完全拉伸跟腱时（保持膝盖伸直），有疼痛的感觉吗？

|  |  |  |  |  |  |  |  |  |  |  |
| --- | --- | --- | --- | --- | --- | --- | --- | --- | --- | --- |

剧烈疼痛 无痛

0 1 2 3 4 5 6 7 8 9 10

3. 当您在平路上行走30分钟后，在随后的2小时内，有疼痛的感觉吗？

（如若因为疼痛无法在平路上行走30分钟，则此题为0分）

|  |  |  |  |  |  |  |  |  |  |  |
| --- | --- | --- | --- | --- | --- | --- | --- | --- | --- | --- |

剧烈疼痛 无痛

0 1 2 3 4 5 6 7 8 9 10

4. 当您以正常步态下楼梯时，有疼痛的感觉吗？

|  |  |  |  |  |  |  |  |  |  |  |
| --- | --- | --- | --- | --- | --- | --- | --- | --- | --- | --- |

剧烈疼痛 无痛

0 1 2 3 4 5 6 7 8 9 10

5. 当您在平地上单脚做10个起踵动作期间或完成后，有疼痛的感觉吗？

|  |  |  |  |  |  |  |  |  |  |  |
| --- | --- | --- | --- | --- | --- | --- | --- | --- | --- | --- |

剧烈疼痛 无痛

0 1 2 3 4 5 6 7 8 9 10

6. 您能在无痛的情况下做几个单脚跳？

|  |  |  |  |  |  |  |  |  |  |  |
| --- | --- | --- | --- | --- | --- | --- | --- | --- | --- | --- |

0 10

0 1 2 3 4 5 6 7 8 9 10

7. 您目前在运动或做其它体力活动吗？

|  |
| --- |

0 完全没有

|  |
| --- |

4 适当的训练和适当的竞技运动

|  |
| --- |

7 重返受伤前的训练水平但仍未达到受伤前的竞技水平

|  |
| --- |

10 达到受伤前一样或更高的竞技水平

8. 请您根据自身情况完成A或B或C的问题

- 如果您在进行需跟腱负重的运动时，没有疼痛的感觉，请做A题
- 如果您在进行需跟腱负重的运动时，有疼痛感但不影响您继续完成运动，请做B题
- 如果您在进行需跟腱负重的运动时，由于疼痛而无法继续下去，请做C题

A. 如果您在进行需跟腱负重的运动时，没有疼痛的感觉, 您能运动多长时间呢？

0 1-10分钟 11-20分钟 21-30分钟 >30分钟

|  |
| --- |

|  |
| --- |

|  |
| --- |

|  |
| --- |

|  |
| --- |

0 7 14 21 30

B. 如果您在进行需跟腱负重的运动时，有疼痛感但不影响您继续完成运动, 您能运动多长时间呢？

0 1-10分钟 11-20分钟 21-30分钟 >30分钟

|  |
| --- |

|  |
| --- |

|  |
| --- |

|  |
| --- |

|  |
| --- |

0 7 14 21 30

C. 如果您在进行需跟腱负重的运动时，由于疼痛而无法继续下去，您能运动多长时间呢？

0 1-10分钟 11-20分钟 21-30分钟 >30分钟

|  |
| --- |

|  |
| --- |

|  |
| --- |

|  |
| --- |

|  |
| --- |

0 7 14 21 30
